# The impact of interpreting students’ gestures and speech content on speech fluency of consecutive interpreting

**DOI:** 10.3389/fpsyg.2025.1568341

**Published:** 2025-05-23

**Authors:** Qiuya Zhang, Youping Jing

**Affiliations:** College of Foreign Languages and Cultures, Xiamen University, Xiamen, Fujian, China

**Keywords:** interpreter, speech content, speech fluency, free gesture, restricted gesture

## Abstract

Gestures, as non-verbal cues, are found to overcome lexical limitations, address grammatical challenges, and improve speech by helping maintain spatial imagery during the lexical search process. Speech content involving spatial imagery tends to elicit greater reliance on gestures. However, little attention was given to exploring the role of interpreting students’ gestures in speech performance, particularly in terms of fluency. This study examined the fluency performance of 17 interpreting students, focusing on their speech rate, average pause length, disfluency rate, and disfluency duration. The interpreting students were asked to complete four consecutive interpreting tasks under two conditions: Free Gesture (F) and Restricted Gesture (R). This study employed an experimental design and conducted post - task interviews to investigate the impact of gestures on the speech fluency performance of interpreting students. The findings indicated that restricting gestures leads to a significant increase in both disfluency duration and disfluency rate among interpreting students. In contrast, there were no statistically significant differences in speech rate or average pause length between conditions. Moreover, when interpreting spatial content, the absence of gestures was associated with further significant increases in disfluency duration and disfluency rate. This indicated that gestures partially facilitate speech fluency, particularly when processing complex spatial information. Additionally, the overall fluency of interpreting students appears to be closely linked to their proficiency in switching languages. These findings highlight the significant role of gestures in enhancing interpreting students’ performance and suggest avenues for further exploration of gestures’ impact on various aspects of interpreting.

## 1 Introduction

Interpreters’ gestures are often regarded as auxiliary rather than primary communication tools. Their role as an essential component of the interpreter’s internal communication is often overlooked (Viaggio, 1997). For speakers, co-speech gestures serve not only as vehicles for communication but also embody a cognitive presence (McNeill, 2000). Co-speech gestures offer insight into the speaker’s mental representations and processes (Goldin-Meadow et al., 1993; Kendon, 2009; McNeill, 1992). Gesture studies identified the supportive role of gestures in speech production, which, according to Levelt (1999), involves three key stages: conceptualization, formulation, and articulation. Empirical evidence showed that gestures positively impacted speech articulation, enhancing content, fluency, length, and prosody (e.g., Cravotta et al., 2019; Finlayson et al., 2003; Jenkins et al., 2017; Kirk and Lewis, 2016; Mol and Kita, 2012; Morsella and Krauss, 2004; Rauscher et al., 1996). Furthermore, gestures contributed to an increased speech rate, reduced disfluency rate, and shorter pauses among speakers, thereby enhancing speech fluency (Finlayson et al., 2003; Ma et al., 2021; Rauscher et al., 1996; Satō, 2020). Research also showed that gestures facilitated the breakdown of complex spatial concepts into simpler, expressible components, enhancing overall speech performance (Finlayson et al., 2003; Rauscher et al., 1996). They were particularly effective in communicating spatial information as they supported the activation and retention of visuospatial images in working memory, reducing the cognitive burden during narrative tasks (Eielts et al., 2018; Kita, 2000; Kita et al., 2017; Morsella and Krauss, 2004). These effects related to tasks also extended to bilingual individuals (Aziz and Nicoladis, 2018; Stam, 2016). The interplay among gestures, spatial content, and speech performance was highlighted by the Information Packaging Hypothesis (IPH), which suggests that gestures help organize complex information like spatial content into manageable verbal units in speech production (Alibali et al., 2000).

Interpreting studies have delved into the importance of interpreters’ speech production with gestures (De León and Santana, 2021; Galvão, 2009, 2013, 2020; Stachowiak-Szymczak, 2019; Vranjes and Brône, 2021). The gesturing process reflects not only individual style and inclination but also cognitive load in speech production (De León and Santana, 2021; Stachowiak-Szymczak, 2019; Vranjes and Brône, 2021). Existing research concludes that interpreters’ gestures serve two functions: promoting discussion in triad communication (Vranjes and Brône, 2021), and facilitating interpreting speech production (De León and Santana, 2021; Galvão, 2013; Stachowiak-Szymczak, 2019). Interpreters’ gestures (e.g., pragmatic gestures) have been found to support their lexical retrieval and reduce cognitive load, thereby facilitating fluent speech production (Galvão, 2013; Stachowiak-Szymczak, 2019). Gestures produced during simultaneous interpreting also serve pragmatic functions or act as self-adaptors to maintain self-focus (Cienki, 2024).

Unlike simultaneous interpreting—where interpreters work in booths and remain unseen—consecutive interpreting occurs after the source-language speech and relies heavily on visible gestural communication (Pöchhacker, 2016). In practice, interpreters often contend with different types of operational constraints (e.g., managing a microphone or a microphone and laptop), which can restrict their ability to gesture freely and, in turn, impair speech fluency. Consequently, limited gesture use may undermine overall performance across various speech tasks.

Existing research has demonstrated that interpreters’ gestures play multifaceted roles in reducing cognitive load and enhancing audience comprehension. However, relatively little attention has been devoted to investigating how gestures specifically affect interpreters’ speech fluency during consecutive interpreting—particularly in situations where interpreters are unable to effectively employ gestures. Moreover, the underlying mechanisms by which gestures influence speech fluency in interpreting remain largely unexplored. This study adopts the IPH in explanation, which aims to explore the influence of interpreting students’ gestures on consecutive interpreting fluency performance in relation to speech content with important practical and theoretical implications. Practically, it raises crucial awareness among interpreting professionals about the challenges posed by limited gestural expression in their work environments. Current interpreting conditions often fail to prioritize creating spaces where interpreters can use gestures freely and emphasize the importance of interpreters’ gesture usage on certain occasions with specific content. Theoretically, this work provides a fresh perspective on the interplay between gestures and interpreting fluency, further extending the IPH to the context of consecutive interpreting, demonstrating that gestures not only facilitate spontaneous speech production but also play a crucial role in structuring and delivering information in high-cognitive-load bilingual tasks.

## 2 Literature review

### 2.1 Gestures and speech fluency performance

Numerous researchers have explored the relationship between gestures and language-specific speech performance, considering the supportive role of gestures in speech production. These studies have investigated how variations in gesturing conditions affect speech measures like fluency (Cravotta et al., 2019; Finlayson et al., 2003; Jenkins et al., 2017; Kirk and Lewis, 2016; Mol and Kita, 2012; Morsella and Krauss, 2004; Rauscher et al., 1996). The results have been inconsistent across studies. Finlayson et al. (2003) manipulated various gesture conditions—both-hand gestures, single-hand gestures, and free gestures—to assess their impact on fluency. They found that speakers prohibited from gesturing with one or both hands experienced a significant increase in disfluency, including filled and silent pauses, repetitions, and reformulations. This highlighted the critical role of gestures in alleviating cognitive load, as reflected in the higher word count observed when speakers were permitted to gesture compared to situations in which gestures were restricted during narrative production. In contrast, Cravotta et al. (2019) found that neither not encouraging nor encouraging gestures affected speech disfluency, including filled pauses, self-corrections, repetitions, insertions, interruptions, or speech rate changes. These findings may be attributed to the study’s design, which compared “not encouraging” versus “encouraging gestures” rather than contrasting naturally occurring gestures with restricted ones. In the “not encouraging gestures” condition, participants could still use gestures naturally. However, when gestures were restricted, participants often felt uncomfortable or struggled to articulate complex concepts, resulting in increased speech disfluency.

Previous research has established a connection between gestures and speech proficiency in the realm of second language acquisition (SLA). Bilingual individuals, when naturally using gestures, exhibited a greater use of word tokens, word types, and scene descriptions in communication compared to situations in which their gesture use was restricted (Laurent and Nicoladis, 2014). Satō (2020) evaluated fluency in terms of speed through the analysis of the average number of syllables per utterance. The results indicated that L2 utterances increased in complexity and fluency when accompanied by gestures. Additionally, Ma et al. (2021) identified a positive correlation between the rate of representational gestures and the number of word types and the speech rate of low and medium proficiency L2 speakers, while also highlighting a negative association with the mean length of pauses. This research explored the connection between gesture usage and L2 speech performance, with a specific emphasis on fluency, suggesting the crucial role of gestures in supporting L2 expression.

Gestures can assist in overcoming fluency challenges during speech production (Kirk and Lewis, 2016; Satō, 2020), highlighting the significant link between speech production and gestures in bilingual contexts. Given that interpreters are unique bilingual professionals who operate under high cognitive demands (Dong, 2023), this correlation may extend to the interpreting context, specifically to the connection between an interpreter’s gestures and interpreting fluency, emphasizing the crucial role of gestures in language production. Emerging research on non-verbal behaviors in interpreting (Nicodemus and Emmorey, 2013) further suggested that gestures play a crucial role in managing language production challenges during real-time tasks.

General bilinguals have varying levels of proficiency in a second language (L2) but lack extensive experience in translation or interpreting tasks (Dong, 2023). Despite the significance of gesture usage, there is a dearth of pertinent research in the field of interpreting. Previous research has explored how speakers’ styles influence the gesture behavior of simultaneous interpreters (Galvão, 2020) and the relationship between interpreters’ gesture frequency and cognitive stress (Stachowiak-Szymczak, 2019). These studies have elucidated key factors such as the speaking style of presenters and the cognitive load linked to interpreters’ gestures, quantified by gesture frequency and type. Existing research has not yet examined the impact on interpreters’ speech production when they are unable to employ gestures effectively. This issue warrants investigation, as interpreters frequently encounter restrictions on gestural expression in various settings. For example, they may be constrained by using handheld microphones or by the simultaneous management of both microphones and notebooks. Understanding the implications of these gestural restrictions could be crucial for enhancing the quality and efficiency of interpreting services.

### 2.2 Speech fluency in interpreting

Fluency, as discussed by Lennon (1990), has both broad and narrow definitions. In a broad context, it is often synonymous with “overall language proficiency” (Chambers, 1997; Lennon, 1990). In its narrow scope, fluency specifically addresses the capacity of L2 learners to articulate their thoughts without unnecessary pauses or hesitations (Skehan, 2009; Tavakoli and Skehan, 2005). This more specific interpretation of fluency, centers on the smooth flow of speech production. Fluency can be assessed in various ways, e.g., in terms of the rate of speaking or the number of breakdowns or repairs (Skehan, 2009).

Speech fluency is crucial for the quality of interpreting. Although it is frequently overlooked in interpreter education and training programs, many scholars have referenced concepts similar to speech fluency in the context of evaluating interpretation quality. For instance, Lee (2008) argued that the quality of interpreting should be assessed based on three dimensions: accuracy and target language quality, and speech delivery. Speech delivery should consider whether interpreters exhibit excellent performance with few deviations, such as inarticulate speech, pauses, hesitation, false starts, fillers, distracting noises, repetition, excessive repairs or self-corrections, unconvincing voice quality, monotonous intonation, and an irritatingly slow speech rate. Han (2015) posited that the standards for evaluating interpreting quality should focus on three dimensions: information completeness (InfoCom), fluency of delivery (FluDel), and target language quality (TLQual).

Fluency has also considered significant for assessing interpreting quality by professional interpreters (Kurz, 2002; Pöchhacker and Zwischenberger, 2010; Zwischenberger, 2010) and listeners (Amini et al., 2013; Rennert, 2010; Yu and Van Heuven, 2017). Moreover, speech fluency serves as a more immediate indicator of changes in interpreters’ cognitive load compared to other factors, enabling interpreters to adjust their cognitive efforts to mitigate major errors and omissions (Gieshoff, 2021).

Mead’s (2005) influential work is one of the few studies that have examined the temporal parameters affecting interpreting fluency. His examination of five temporal parameters indicated that speech rate, pause duration, and length of fluent sequences are essential elements in evaluating fluency. Lin et al. (2018), Yang (2018), and Yu and Van Heuven (2017) have identified valid and reliable measures for interpreting fluency, including speech rate, pauses, repetitions, restarts, false starts, corrections, mean length of run (MLR), speech time, and phonation time ratio. Our study examines the impact of gestures on interpreters’ speech fluency by comparing conditions in which gesturing is restricted—specifically when interpreters must hold a notebook—with conditions where gesturing remains unrestricted. Building on previous findings that highlight the positive effects of gesturing on speech production, particularly fluency (Alibali et al., 2000; Rauscher et al., 1996), we analyze fluency using multiple measures. These include speech rate (the rate of fluent speech), mean pause length (an indicator of fluency breakdown), disfluency rate and disfluency duration (encompassing self-corrections, repetitions, and lengthening).

### 2.3 Gestures, interpreting fluency, and the information packaging hypothesis

The IPH, originally proposed by Kita (2000), suggests that gestures are not just add-ons to speech in facilitating comprehension but deeply involved in the conceptual planning process that can benefit the speaker more directly. This hypothesis suggests that gestures aid speakers in structuring information into verbal units. They help speakers break down complex content into smaller, easier-to-express parts (Alibali et al., 2000). This packaging procedure is especially important when the information to be expressed is difficult to conceptualize.

In consecutive interpreting, the cognitive load is rather high (Stachowiak-Szymczak, 2019). Interpreters need to quickly and accurately interpret the source language into the target counterpart. Hence, in this procedure, gestures can serve as a useful cognitive tool to reduce cognitive load (Galvão, 2013; Stachowiak-Szymczak, 2019). For instance, gestures can help to break down and structure complex information like spatial details in the source language (Hostetter et al., 2007) for verbal production in target language. Additionally, while some theories (e.g., Lexical Access Hypothesis) argue that gestures directly assist in word-finding (Chawla and Krauss, 1994; Krauss et al., 2001), the IPH extends their ideas and suggests that gestures also facilitate the retrieval of complicated information while assisting in conceptualization (Baddeley, 1986) in target language before words are chosen in interpreting. This procedure can be beneficial for interpreters who need to manage and recall large amounts of information in real time. Thus, by offloading some of the conceptual processing to gestures, interpreting with gestures helps lighten the cognitive load.

When interpreters are required to restrict their natural gestures—for instance, by holding a notebook during interpretation—their ability to execute complete gesture units (including preparation, stroke, and retraction as described by Kendon, 1980) is compromised. Although some minimal hand movement may occur, this restriction limits the production of complete gestures, especially representational, and iconic gestures, which have been shown to play a critical role in conceptualization from the perspective of the IPH (Hostetter and Alibali, 2004; Hostetter et al., 2007). Consequently, such gesture-restricted conditions may adversely affect both speech production and overall interpreting quality—especially the interpreter’s ability to produce smooth and coherent speech. Few studies have specifically examined the impact of restricting natural gesture use on interpreting fluency, nor have they adequately addressed the underlying mechanisms. This gap emphasizes the need for further research to elucidate the cognitive and linguistic processes through which full-range, natural gestures facilitate speech fluency in interpreting, as well as to develop strategies that counteract the adverse effects of restricted gestural expression.

This study uses four fluency indicators from previous research (Alibali et al., 2000; Rauscher et al., 1996)—speech rate, mean pause length, disfluency rate, and disfluency duration—to explore the role of gestures in speech production. These measures help capture different aspects of fluency in verbal communication (Segalowitz, 2010). Research suggests that fluency indicators vary in how they relate to language proficiency. Speech rate and pause patterns tend to have stronger links to language skills (Dörnyei and Kormos, 1998; Segalowitz, 2010), likely because more advanced speakers can control speech rhythm more effectively using automatic language processes (Dörnyei and Kormos, 1998). Bortfeld et al. (2001) found that disfluency rates rise significantly when speakers describe abstract figures, and these disfluencies follow different timing patterns than pauses. This suggests that disfluencies mainly reflect cognitive load during speech planning, including real-time monitoring, word retrieval, and self-correction. Pauses, on the other hand, may act as planning buffers rather than direct signs of mental strain. From the perspective of the IPH, limiting gestures in interpreting may disrupt fluency in two ways. First, without gestures to support thought organization, interpreters must rely more on verbal strategies to restructure information, which increases cognitive strain. Second, while proficient second-language speakers can adjust speech rate and pauses to maintain fluency (Dörnyei and Kormos, 1998), these strategies may not be enough to prevent disfluencies caused by difficulties in conceptualization. This distinction highlights the different cognitive processes behind various fluency indicators. However, little research has explored how gesture restriction affects these fluency measures and their relationships under high cognitive load during interpreting.

The IPH posits that multimodal communication—particularly the coordination of speech and gestures—optimizes the transmission of complex information, especially spatial content (Kita, 2000; Kita et al., 2017). Empirical evidence demonstrates that gestures reduce cognitive load during narrative tasks by activating and sustaining visuospatial representations in working memory (Eielts et al., 2018; Morsella and Krauss, 2004). This facilitative effect exhibits content-specific patterns: When describing spatial scenarios (e.g., a coyote devising strategies to outsmart a roadrunner in animated narratives), speakers permitted to gesture exhibit faster speech rates and fewer intra-clausal filled pauses compared to those under gesture restriction (Rauscher et al., 1996). Crucially, this advantage disappears in non-spatial discourse, suggesting gesture’s specialized role in spatial conceptualization.

Notably, these effects extend to bilingual populations. Second language (L2) learners produce significantly more iconic gestures during cognitively demanding tasks like cartoon retelling than in structured interviews (Aziz and Nicoladis, 2018; Stam, 2016). For professional interpreters—a unique bilingual cohort requiring intensive linguistic and cultural mediation—the absence of gestural scaffolding may impose heightened cognitive strain. Unlike general bilinguals, interpreters must manage precise lexical retrieval, pragmatic adaptation, and rapid language switching under time constraints, factors known to amplify processing demands and psychological stress (Ferreira and Schwieter, 2023). Despite gesture’s established role in mitigating cognitive load, critical gaps persist regarding how gesture restriction differentially impacts interpreting fluency across spatial versus non-spatial content, and whether interpreters’ specialized training enables compensatory strategies absent in typical L2 speakers. Resolving these questions holds theoretical implications for the IPH in the underexplored field of interpreting and practical significance for interpreter pedagogy.

## 3 Methodology

### 3.1 Research questions

The research questions are: (1) When the interpreter students’ gestures are restricted, is their interpreting fluency—measured by speaking speed, average pause length, and the rate and duration of disfluencies—affected compared with the situation when they can gesture naturally? (2) If yes, is the drop in fluency even greater when handling complex information (spatial details)? If no, why?.

### 3.2 Participants

The study involved 17 postgraduate students who were enrolled in the interpreting program at one of China’s First-Class Universities. These students had completed their first year of interpreting studies, which entailed nearly 9 months of intensive training. All participants had undergone 2 interpreting training courses during their undergraduate studies. They had acquired various interpreting skills, such as retelling, note-taking, consecutive interpreting with note-taking, and some had also received training in memorization and consecutive interpreting without note-taking. They typically used notes for consecutive interpreting, translating moderate information density speeches spoken at a normal pace, defined as a speaking rate of 250–450 ms per syllable in Mandarin (Wu and Zhu, 2001), and shorter segments, especially in contexts like business negotiations and training seminars. The sample size (*N* = 17) was constrained by practical limitations in recruiting interpreting students. Seventeen participants randomly completed 4 interpreting tasks (2 with gestures, 2 with restricted gestures). Consequently, the data quantity for both the gesture and restricted-gesture conditions was 34 each (17 participants × 2 tasks).

The participants, all right-handed females, had an average age of 23.18 years (Min = 22, Max = 29, SD = 1.63). Each student designated Chinese as Language A and English as Language B (namely, these students were asked to interpret Chinese into English). Before commencing the experiment, the participants underwent assessments to ensure uniformity in their interpreting proficiency levels. Two interpreter trainers, each with at least 2 years of experience in interpreting and teaching, assessed the interpreting skills of the 17 participants. The assessment adhered to the scoring standards and methodology of the China Accreditation Test for Translators and Interpreters (CATTI). CATTI, administered by the National Translation Test and Appraisal Center, is the sole language examination integrated into China’s national vocational qualification system (CATTI Center, 2023). Each participant’s interpreting performance was independently evaluated by two assessors who were provided with a recording of identical content from a randomly selected participant. The final score for each participant was determined as the average of the ratings given by the two assessors. The assessment utilized a five-point rating scale (1–5) with categories including Excellent, Good, Average, Pass, and Fail. The inter-rater agreement exhibited a high level of consistency, with a Cohen’s Kappa coefficient of 0.81 and a *p*-value below 0.01. The results indicated that all 17 participants demonstrated proficiency in autonomously engaging in moderately demanding interpreting tasks (*M* = 3.59, SD = 0.54).

### 3.3 Materials

The study utilized four Chinese texts chosen through a rigorous process to ensure diversity. Initially, six texts were sourced from the internet, with three focusing on travel introductions and the remaining three on scientific experiments. Subsequently, a native Chinese researcher with expertise in linguistics was enlisted to edit the texts, ensuring consistency in Chinese expression. To ensure consistency in the use of Chinese, two independent researchers followed these steps. First, specialized terms—technical, academic, or field-specific—were eliminated to maintain clarity. Second, the tone, voice, and language style were standardized to ensure uniform use of formal and informal language as appropriate. Third, the materials were reviewed for grammatical accuracy and adherence to standard Chinese typographical conventions. Finally, the edited materials were refined to ensure they were error-free and easily comprehensible.

Subsequently, two authors cross-reviewed each other’s work and engaged in discussions. If any conflicting opinions arose, an interpreting expert was invited to review the material and join the discussion until consensus was reached among all three parties. This standardization process aimed to enhance comparability among the six texts and streamline subsequent analyses.

Following selection, the six texts underwent evaluation using Chi-Editor to assess their lexical difficulty, text length and text difficulty, grading, as outlined in Table 1. Chi-Editor is an online assessment tool created to evaluate Chinese reading texts and align them with the proficiency levels specified in the International Curriculum for Chinese Language Education (Hanban, 2014). This evaluation process involves annotating texts based on various lexical and syntactic attributes, providing valuable insights to assess text complexity, and assisting in lexical and syntactic annotation during text adaptation.

**Table 1:** Text analysis results using Chi-Editor and CRIE 3.0.

Number of text	Lexical difficulty	Text length	Text difficulty	Grading	Density of propositional words
Text 1	0.26	586	2.93	Medium level 4	0.77
Text 2	0.28	575	2.91	Medium level 4	0.79
Text 3	0.28	574	2.95	Medium level 4	0.77
Text 4	0.27	577	2.99	Medium level 4	0.79

Subsequently, the six texts underwent evaluation using CRIE 3.0 (Chinese Readability Index Explorer), a tool developed by the Readability Research Group (Fu-Yuan et al., 2018; Lin et al., 2019; Tseng et al., 2019). This step complemented the capabilities of Chi-Editor, as Chi-Editor does not analyze density of proposition words (see Table 1).

Two experts were assigned the task of evaluating the difficulty of interpreting the six texts from various perspectives, aiming to select four texts with similar difficulty levels. The experts assessed the interpreting challenges posed by the six texts to students who had completed their first year of postgraduate study with a nine-month interpreting course. The evaluation process was based on nine criteria, including the following: (1) overall difficulty; (2) word difficulty; (3) syntactic difficulty; (4) information density; (5) abstraction; (6) logic; (7) clarity; (8) coherence; and (9) difficulty with knowledge (Liu and Chiu, 2009). Two experts independently evaluated six interpreting tasks, demonstrating strong inter-rater reliability with a Spearman’s rho coefficient of 0.65 (*p* < 0.01). Based on this consistent assessment, four texts of comparable difficulty were selected as experimental materials.

Following the text selection process, a native Chinese speaker recorded videos of the four experimental texts and two practice texts. The speaker delivered the content naturally at a moderate pace of 240–280 words per minute (Wu and Zhu, 2001), demonstrating standard pronunciation, accurate vocabulary, and natural intonation, with only occasional minor errors in tone or articulation. Gestures were allowed freely during recording without restrictions. To ensure consistency across conditions, a Kruskal–Wallis test—a non-parametric statistical analysis was conducted and revealed no significant differences in gesture frequency [entire gesture units were counted (Kendon, 1980)] among the videos (*p* > 0.05), confirming uniformity in experimental settings and reducing potential research bias.

Lastly, the recorded videos were imported into Audacity for post-processing, including activities like noise reduction and video segmentation. The video has a total duration of 3 min, split into three roughly equal segments of 1 min each. This arrangement enables interpreters and interpreter trainees to establish the duration they can manage without note-taking (Stachowiak-Szymczak, 2019).

### 3.4 Procedure

All study participants had to complete an informed consent form before entering individual testing rooms in sequence. The experimental protocol was clearly explained to each participant. Participants were required to complete four consecutive interpreting tasks from language A (Chinese) to language B (English).

In professional interpreting environments, interpreters usually receive conference materials from the organizer. To replicate this real-world scenario, participants in the study were briefed on the text topics and provided with a list of relevant vocabulary the day before the experiment started.

The tasks were arranged using a Latin square design, which created an 4*4 Latin square matrix. This design ensures that each treatment combination appears uniquely in every row and column, effectively eliminating confounding factors by pairing each treatment level equally with others. Before the task they completed two brief video trials to become familiar with the experimental procedures.

Seventeen participants were randomly assigned to one of two conditions in experiments: Free Gesture (F) and Restricted Gesture (R). In the Restricted Gesture condition, participants were asked to hold a microphone in one hand and a notebook in the other. This setup ensured ecological validity by closely mirroring the constraints interpreters often face in professional settings. In the Free Gesture condition, participants’ natural gestural behavior was neither encouraged nor discouraged. Our observations through direct observation and video recordings indicated that the majority of interpreters permitted to use gestures did utilize them. All participants were instructed to stand within a designated area, ensuring their feet remained within a specified zone to stay within the camera’s view. Participants were informed that any foot movement could compromise the quality of the recording, thereby ensuring that any observed disfluency was attributed to restricted gesturing rather than limited motor movement.

To prevent fatigue, interpreters were provided with adequate rest periods after each task. After completing all four interpreting tasks, each participant would participate in a post-test interview to investigate their subjective perceptions of gestural communication. Thematic analysis centered on participants’ perceptions of two critical factors influencing interpreting fluency: (1) the role of co-speech gestures and (2) the impact of source speech content.

In the experiment’s speaker videos, interpreters had access to the speaker’s upper body and gestures. A Latin Square design was employed to balance experimental conditions and minimize bias, ensuring equal representation of each condition and preventing any single variable from disproportionately influencing the results. This design allowed for a more reliable attribution of differences in interpreters’ performances to the experimental conditions rather than individual speaker characteristics. During the experiment, interpreters engaged in consecutive interpreting without using written scripts or taking notes. This type of consecutive interpreting, which does not involve note-taking, is sometimes called “brief” consecutive interpreting, as opposed to “traditional” counterpart that includes note-taking, often used in liaison settings with a bidirectional approach (Pöchhacker, 2016).

To ensure ecological validity, the experiment replicated real-world consecutive interpreting conditions. Interpreters performed in the same physical space as both the speaker and a single audience member (the experimenter), mirroring typical settings where all participants can observe each other’s gestures and expressions (Bühler, 1985). The audience seating was intentionally arranged to allow the interpreter to naturally see both the speaker and the experimenter, as restricted visibility could distort performance. To minimize artificial effects, interpreters were not told the experimenter would act as the audience, avoiding potential bias from knowing their dual role. Using only one audience member maintained experimental consistency while reflecting common real-world scenarios where interpreters engage with unfamiliar listeners lacking shared background knowledge. After the experiment, we conducted a brief semi-structured interview with each participant to gather additional qualitative insights. The interview included two questions: (1) Does holding an object in your hand affect your interpreting performance? If so, what specific effects does it have on your performance? and (2) Which of the four translation pieces do you find most challenging, or which ones do you find difficult, and what are the specific challenges you encounter? After completing the interview, each participant received a monetary compensation of 60 RMB for their time and effort.

Each sound segment was captured and recorded using a PHILIPS Voice Tracer. To improve audio quality and minimize environmental interference, the Voice Tracer device was securely attached to the participant’s collar, ensuring it stayed within the lips’ reception range.

### 3.5 Data collection and analysis

Following automated transcription of all 68 recordings (17 participants * 4 texts) using PHILIPS Voice Tracer’s speech-to-text software, the first author thoroughly checked each transcript against the original audio to ensure accuracy. The second author reviewed and corrected any inaccuracies or omissions in the machine-generated text, ensuring that filled pauses and speech disfluencies were preserved for precision.

Each of the 68 audio files was divided into two sections, creating two files per text for easier annotation. The files were organized into folders based on the experimental conditions: Free Gesture or Restricted Gesture. This organization facilitated more efficient annotation and data analysis.

Tavakoli and Skehan (2005), assessed through speed rate (the rate of fluent speech), the breakdown of fluent speech (mean length of pauses), and disfluency rate and duration. The chosen fluency metrics for this study included:

- Speech rate (syllables/s): calculated as the total number of syllables divided by the overall speech duration (Rauscher et al., 1996; Satō, 2020; Yang, 2018; Yu and Van Heuven, 2017);
- Mean length of pauses (in seconds): determined by dividing the total pause duration (in seconds) by the number of pauses (Ma et al., 2021; Yu and Van Heuven, 2017);
- Disfluency rate (disfluent words/min): calculated as the number of repair disfluent words (covering self-corrections, repetitions, and lengthening) divided by the overall speech duration (in minutes) (Ma et al., 2021; Rauscher et al., 1996);
- Disfluency duration: total duration (in seconds) of disfluent speech. The sentence was transcribed as “…you know the transparent uhm transportation and absorption of water in a plant…” While interpreting, the interpreter substituted “transparent” with “transportation,” identifying it as a self-correction of a disfluency. The duration taken for this correction will be regarded as the disfluency duration.

The Syllable Counter – WordCalc tool was utilized to determine the syllable count for each interpreter in the transcription. The PRAAT software was used to automatically mark silent pauses by executing the mark-pauses script, developed by Lennes (2002). This script detected silent pauses in the extended audio segments exceeding 0.25 s, integrated them into the Text Grid interval, and indicated the duration of each pause that surpassed the specified threshold. Silent pauses were measured in milliseconds, with a sampling threshold set at 59 dB as the maximum intensity. A typical threshold of 0.25 s for silent pauses was commonly utilized to differentiate between speech hesitations and pauses inherent in the normal articulation process, or those that may be considered as micro-pauses (Pinget et al., 2014; Yu and Van Heuven, 2017). The first author subsequently conducted a secondary verification of the silent pauses identified by the algorithm to ensure the accuracy of the annotations. Concurrently, an interpreting instructor with 3 years of experience in interpreting education meticulously annotated filled pauses and other disfluencies. Repair disfluencies, including repetitions, self-corrections, lengthening, and irrelevant words related to the speech tasks, were annotated in the transcription for analysis.

Moreover, words with spatial concepts in four experimental texts were recognized and counted in each sentence. In Chinese locative expressions, spatial relationships between figure and ground were depicted not only through prepositions but also through localizers (Hsiung, 2024). We identified all phrases in the texts containing spatial prepositions and localizers. This article used Weiciyun’s Chinese analysis feature to statistically analyze localizers and prepositions in the text. It employed the Jieba algorithm, a leading Python module for Chinese text segmentation. Jieba segments text using dictionary matching and a maximum matching method, enhanced by the Hidden Markov Model (HMM) for greater accuracy. The Chinese lexicon used for segmentation was based on the People Daily corpus, Peking University’s Institute of Computational Linguistics, Baidu, and private lexicons, as well as a stop-word lexicon from sources including the Harbin Institute of Technology and Baidu.

## 4 Results

The study included both quantitative and qualitative data. The first section assessed interpreting fluency, including speech rate, average pause length, disfluency rate, and disfluency duration across different gesture conditions. The second section of the study looked at post-task questionnaires to understand interpreters’ views on factors causing difficulty of interpreting fluency.

### 4.1 Statistical analysis

#### 4.1.1 Interpreters’ speech fluency in free gesture and restricted gesture conditions

This study investigated the impact of gestures on language fluency by comparing fluency measures under the Free Gesture and the Restricted Gesture conditions. According to the data presented in Tables 2, 3, the primary fluency indicators include speech rate, mean length of pauses, disfluency rate, and disfluency duration. Additionally, since all text contents were balanced in the experiment to ensure that the difficulty and complexity of the texts remained consistent across different conditions, this measure reduced the impact of the text content itself on interpreting fluency, ensuring that differences under gesture conditions primarily stemmed from the use of gestures.

**Table 2:** Descriptive statistics of fluency measures for Free and Restricted Gestures.

Fluency measures	Free gesture			Restricted gesture		
	*M*	SD	IQR	*M*	SD	IQR
Speech rate	3.20	2.25	2.66	3.26	2.68	2.05
Mean length of pauses	0.58	0.14	0.18	0.64	0.26	0.21
Disfluency rate	3.92	1.77	2.25	5.22	2.03	3.04
Disfluency duration	12.31	6.03	9.17	16.90	7.45	9.67

Mean (M), standard deviation (SD), and interquartile range (IQR) for fluency measure calculations.

**Table 3:** Variance analysis of the impact of gesture usage on interpreting fluency performance.

Fluency measures	*F*	*P*-value	Partial eta squared (partial η^2^)
Speech rate	0.013	0.910	0.000
Mean length of pauses	1.352	0.249	0.020
Disfluency rate	8.030	0.006	0.108
Disfluency duration	7.779	0.007	0.105

In terms of speech rate, participants using the Free Gesture exhibited an average of 3.20 (SD = 2.25, IQR = 2.66), while the average speech rate under the Restricted Gesture was 3.26 (SD = 2.68, IQR = 2.05). These data did not reach statistical significance (*F* = 0.013, *p* = 0.910).

Regarding mean length of pauses, the average for the Free Gesture condition was 0.58 (SD = 0.14, IQR = 0.18), whereas the average for the Restricted Gesture condition was 0.64 (SD = 0.26, IQR = 0.21). Similarly, this measure did not achieve significance (*F* = 1.352, *p* = 0.249), but the shorter pauses associated with the Free Gesture might indicate that gestures help participants express themselves more fluently and reduce unnecessary pauses.

The comparison of disfluency rate revealed that the disfluency rate under the Free Gesture was 3.92 (SD = 1.77, IQR = 2.25), while under the Restricted Gesture it was 5.22 (SD = 2.03, IQR = 3.04). This difference was statistically significant (*F* = 8.030, *p* = 0.006), with a partial *η*^2^ value of 0.108, indicating a medium effect of gesture use on disfluency rate [0.01 is considered a small effect, 0.06 a moderate effect, and 0.14 a large effect (Cohen, 1988)]. This suggested that the Free Gesture might effectively reduce participants’ disfluency, enhancing their overall language fluency.

In terms of disfluency duration, the average for the Free Gesture was 12.31 (SD = 6.03, IQR = 9.17), while under the Restricted Gesture it was 16.9 (SD = 7.45, IQR = 9.67). This difference also reached statistical significance (*F* = 7.779, *p* = 0.007), with a partial *η*^2^ value of 0.105, indicating that variations in gesture conditions significantly impacted disfluency duration. The shorter disfluency duration further corroborated the positive effect of the Free Gesture on fluency, suggesting that gestures might alleviate cognitive load, allowing participants to express themselves more confidently.

Due to multiple comparisons, it was necessary to adjust the significance level. The original significance level was set at 0.05, while the Bonferroni-adjusted significance level needed to be adjusted based on the number of comparisons. In this study, the *p*-values for disfluency rate and disfluency duration were 0.006 and 0.007, respectively. According to Bonferroni’s correction, results were considered statistically significant only if the *p*-value was less than 0.0125. Since the *p*-values for disfluency rate (*p* = 0.006) and disfluency duration (*p* = 0.007) were both less than the adjusted significance level, these two indicators were still deemed significant, indicating that gesture conditions had a significant impact on fluency.

In summary, the study’s findings indicated that the Free Gesture condition significantly enhanced participants’ interpreting fluency—most notably through reductions in disfluency rate and disfluency duration—while gestures did not have a significant effect on speech rate or mean pause length.

#### 4.1.2 Correlation between disfluency rate, disfluency duration, and spatial content

Table 4 presents Pearson correlation results examining associations between disfluency measures and spatial content in discourse. Analysis revealed a weak but statistically significant positive correlation between disfluency rate and spatial content (*r* = 0.206, *p* < 0.001), indicating that increased discussion of spatial content tended to co-occur with higher disfluency rates. Similarly, disfluency duration showed a significant positive association with spatial content (*r* = 0.179, *p* < 0.001), suggesting longer disfluencies occurred more frequently during spatial content elaboration.

**Table 4:** Pearson correlation analysis between disfluency rate, disfluency duration and spatial content.

Metric	Statistic	Disfluency rate	Disfluency duration
Spatial content	Pearson correlation	0.206	0.180
	*p*-value	0.000	0.000
	*N*	634.000	634.000

### 4.2 Interview results

#### 4.2.1 Participants’ perception of the effect of gestures on interpreting speech fluency

From the semi-structured interviews with the 17 participants, it emerged that 13 participants believed gestures could influence their interpreting performance. Four participants believed that there were some benefits to the restricted gesture condition. They noted that the restricted use of gestures could reduce interpreter anxiety and provide reassurance (Participants 1 and 5), thereby enhancing comfort levels and potentially improving translation fluency (Participants 8 and 13).

However, most participants pointed out the negative effects of the restricted gesture condition. They observed that restricted gestures might hinder fluency by limiting expression, obstructing the conveyance of speaker emotions, and increasing pressure on the interpreter (Participant 4). Additionally, restricted gestures could divide attention and elevate cognitive stress, while free gestures support memory consolidation and improve comprehension, ultimately benefiting fluency (Participant 9).

Several participants emphasized that the Free Gesture condition positively influenced interpreting fluency by reducing distractions, lowering cognitive stress (Participants 7, 9, 10, 16). Gesture freely aided comprehension of speech content through reducing distractions (Participant 15), enhancing memory retention (Participants 9, 11, 14), and facilitating effective message delivery (Participant 4).

#### 4.2.2 Participants’ perception of the effect of spatial content on interpreting speech fluency

Participants emphasized that high spatial content that they regarded as “most challenging” interpreting pieces, benefited from the use of gestures, which enhanced the fluency of interpreting. For content with high spatial demands, they believed that interpreting fluency depended on proactive strategies: anticipating comprehension challenges, using mental imagery to process spatial information, and guiding listeners’ understanding before potential misunderstandings emerge, with gestures enhancing the communication of complex spatial details (Participants 11 and 10). Moreover, gestures were particularly beneficial in interpreting tasks involving spatial or specific information, aiding in the retention of event sequences, relationships, and geographical details (Participant 14). Therefore, gestures were deemed essential for clarifying spatial information, which directly impacted interpreting fluency by reducing repeated information and comprehension difficulties (Participant 11).

## 5 Discussion

This study aimed to explore how gestures impact fluency during consecutive interpreting. It also examined whether the influence of gestures on fluency changes depending on spatial content.

This study used four fluency indicators —speech rate, mean pause length, disfluency rate, and disfluency duration—to explore the role of gestures in speech production. The findings partially revealed that gesture use could have a significant impact on fluency. Specifically, in the Free Gesture condition, there was a lower rate of disfluencies (speech errors) and shorter durations of these disfluencies compared to the Restricted Gesture condition. However, there was no significant difference in the average pause length and speech rate between the Free Gesture and Restricted Gesture conditions.

Consistent with existing literature, the present study corroborates the crucial role of gestures in enhancing speech fluency (e.g., Finlayson et al., 2003; Ma et al., 2021; Rauscher et al., 1996; Satō, 2020). Finlayson et al. (2003) posited that gestures supported verbal expression, particularly when memory or spatial conceptualization was involved. Ma et al. (2021) and Satō (2020), focusing specifically on second language (L2) learners, highlighted the importance of gestures in improving L2 speakers’ fluency and speech rate, suggesting that gestures benefited not only native speakers but also significantly aid language learners in verbal production. However, in contrast to previous findings, Finlayson et al. (2003) and Rauscher et al. (1996) noted that restricting gesture use led to increased pauses and repetitions in speech. Our study captured different facets of fluency in verbal communication: the results indicated that when interpreters’ gestures were restricted, both the duration and rate of disfluencies increased. Bortfeld et al. (2001) found increased disfluency rates when speakers described abstract figures, with timing patterns distinct from simple pauses. This suggests disfluencies reflect cognitive effort during speech planning, including real-time monitoring, lexical access, and self-correction. This increased cognitive load during speech planning likely contributes to the elevated disfluency rate and extended disfluency duration observed in gesture-restricted conditions. These observations could align with the Information Packaging Hypothesis (Kita, 2000), which argues that gestures play a critical role in the speaker’s internal conceptualization, going beyond mere comprehension facilitation. The hypothesis posits that gestures assist speakers in organizing information into coherent verbal units, simplifying complex content into more expressible segments (Alibali et al., 2000). This packaging mechanism is particularly vital when expressing conceptually challenging information. These functions of gesture may explain why a gesture-restricted condition leads to a higher disfluency rate and longer disfluency duration.

To further investigate the significant influence of restricted gestures on fluency, specifically whether they contributed to increased disfluency in relation to the speaker’s spatial content, the study conducted Pearson correlation analyses to assess the relationships among disfluency rate, disfluency duration, and spatial content. The results indicated a statistically significant positive correlation between disfluency rate, duration and spatial content. The IPH suggests that the integration of speech and gestures in multimodal communication plays a crucial role in enhancing the clarity and effectiveness of conveying complex information, particularly spatial content (Kita, 2000; Kita et al., 2017). Research has shown that gestures can reduce cognitive load during storytelling tasks by engaging and maintaining visual–spatial representations in working memory (Eielts et al., 2018; Morsella and Krauss, 2004). From the perspective of the IPH, restricting gestures during interpreting may interfere with fluency in two primary ways. Firstly, without the support of gestures for organizing thoughts, interpreters may need to depend more heavily on verbal techniques to restructure information, which can heighten cognitive demands. Secondly, although skilled second-language speakers are capable of modifying their speaking pace and pauses to maintain fluency (Dörnyei and Kormos, 1998), such strategies may still fall short of addressing the disfluencies that arise from challenges in conceptualization. As Viaggio (1997) highlighted, gestures help interpreters maintain coherence with intonation and thought processes, ensuring a smooth flow of communication. Gestures are considered integral to fluent interpreting, aiding in the clear expression of communicative intent. In our study, interpreters were required to constrain their natural gesturing, such as by holding a notebook during interpretation, which impeded their capacity to perform complete gesture units (comprising preparation, stroke, and retraction, as defined by Kendon, 1980). While minor hand movements were still possible, this restriction significantly hindered the production of full gesture units, particularly representational and iconic gestures, which, from the perspective of the IPH, have been demonstrated to play a crucial role in conceptualization (Hostetter and Alibali, 2004; Hostetter et al., 2007). As a result, such gesture-restricted conditions had a detrimental impact on interpreting quality, specifically undermining the interpreters’ ability to deliver smooth and coherent speech. As noted by most interpreters in post-experiment interviews, the restricted gesture condition negatively impacted fluency. Participants indicated that limited gestures hindered expression, obstructed emotional conveyance, and increased pressure on interpreters whereas free gestures enhanced memory consolidation. Additionally, free gestures facilitated thinking and organizing information, allowing for better planning of how to express one’s ideas. These findings further substantiate the detrimental effects of gesture constraints on interpreters’ linguistic fluency.

Conversely, our study indicated that the use of gestures did not necessarily lead to shorter pauses or a higher syllable production rate for interpreters. Our finding contrasted with those of Morsella and Krauss (2004), who reported that speech rate, measured in syllables per second, significantly decreased when participants’ hand movements were restricted compared to when they were free to gesture. Additionally, Rauscher et al. (1996) found that gestures enhanced fluency. Restricting gestures led to an increase in filled pauses, as limiting hand movements made it harder for speakers to retrieve words. This effect was attributed to the association of certain gestures with specific concepts, such as spatial ones. The results also differed from findings in second language (L2) learning research. Satō (2020) demonstrated that L2 utterances were more fluent when gestures were used. The rate of fluent speech, measured by the average number of syllables per utterance, was notably enhanced by gestures across various task types. Ma et al. (2021) further examined the relationship between gesture usage and linguistic performance across various tasks. They found a significant correlation between representational gestures and fluency metrics, such as speech rate and pause duration. Several factors may explain these discrepancies. First, speech rate and pause patterns tend to have stronger links to language skills (Dörnyei and Kormos, 1998; Segalowitz, 2010), likely because more advanced speakers can control speech rhythm more effectively using automatic language processes (Dörnyei and Kormos, 1998). Second, while interpreters utilize gestures to emphasize meaning and serve communicative or pragmatic purposes (Cienki, 2024; Robinson and Ellis, 2008), interpreting tasks are often time-constrained. To maintain fluency under such conditions, interpreters may prioritize clarity and brevity, even when gestures are restricted. For instance, they might avoid detailed descriptions or adopt more abstract language to ensure smooth delivery (Seeber, 2011). Consequently, restricted gestures condition may have little impact on interpreters’ speech rate or pauses.

## 6 Conclusion

This study employed a mixed-methods approach, integrating both quantitative and qualitative methodologies. The findings reveal that gestures significantly influence consecutive interpreting fluency, particularly in terms of disfluency rate and duration. Moreover, these fluency indicators were markedly elevated when interpreter students processed spatial content compared to non-spatial content.

These results contribute to the field of interpreting education, training, and research by examining how gestures and speech content influence interpreting fluency especially offering valuable insights into how non-verbal elements influence interpreting performance. The study highlighted how restricting gestures can significantly impact interpreters’ fluency. Therefore, future interpreting training should include scenarios where students face limitations on gesture use. Moreover, this finding has implications for conference organizers and interpreting professionals, who should recognize the importance of allowing unrestricted gesture use in interpreting settings.

Theoretically, our findings indicate that interpreter students’ gestures embodied this cognitive repackaging process, thereby validating the IPH within professional interpreting contexts and broadening the hypothesis’s applicability to challenges in bilingual communication.

One limitation of this study is its small sample size. *A priori* power analysis was conducted using G*Power (Faul et al., 2007). Based on the design for moderate effect sizes (*f* = 0.25), with *α* = 0.05, power = 0.80, the analysis indicated a required sample size of *N* = 34. A key limitation stems from having only female participants in this study. Since no male interpreters were included, we cannot confirm whether these findings apply equally to men in similar training programs. Although we have mitigated the limitations of the small sample size through carefully designed experimental tasks and rigorous experimental controls, we recognize that the relatively small sample size may have increased the risk of Type II errors (i.e., failing to detect an actual existing effect) and may have affected the generalizability of the results. Future studies can validate and extend the current findings by increasing the sample size. Additionally, our research design did not control for potential confounding factors, namely, participants’ pre-existing gestural experience (e.g., habitual gesture use in daily communication) and individual differences in baseline cognitive load capacity. Future research should implement systematic controls for participants’ gestural experience through pre-experiment screening and incorporate cognitive load measurements (e.g., dual-task paradigms or physiological monitoring) to strengthen the validity of conclusions about gesture restriction effects on speech production. Moreover, although participants held a microphone in one hand and a notebook in the other, this arrangement did not eliminate all hand movements. Pragmatic gestures (e.g., beats) or minor self-adjustment actions (e.g., adjusting the microphone or eyeglasses) might still occur. While our focus was on the impact of restricted versus unrestricted hand gestures on fluency, these residual movements could have partially influenced speech fluency. Future research should address this issue by further refining the experimental setup. In addition, the direction of interpretation (from A to B) may have affected the results, as interpreters’ fluency and processing strategies can vary with interpretation direction (Su and Li, 2019). Therefore, replicating the experiments in the reverse direction (from B to A) is recommended to further validate the present findings. While the observed correlations between spatial content and both disfluency rate (*r* = 0.187) and duration (*r* = 0.151) reached statistical significance (*p* < 0.001), their modest magnitudes suggest limited explanatory power. The weak effect sizes imply that spatial content accounts for only a small proportion of variance in disfluency measures, highlighting the likely influence of unexamined factors—such as individual cognitive strategies, task complexity, or linguistic proficiency—that may mediate this relationship. Although the consistent positive directionality of these associations aligns with cognitive load theories, the practical significance of such weak correlations remains uncertain. These findings underscore the need for caution in interpreting the functional link between spatial content processing and disfluency patterns, as well as the importance of incorporating multimodal measures (e.g., gaze behavior, working memory) in future investigations to disentangle the underlying mechanisms.

## Data Availability

The raw data supporting the conclusions of this article will be made available by the authors, without undue reservation.
